# Real-Time Imaging of DNA Damage in Yeast Cells Using Ultra-Short Near-Infrared Pulsed Laser Irradiation

**DOI:** 10.1371/journal.pone.0113325

**Published:** 2014-11-19

**Authors:** Estrella Guarino, Gheorghe Cojoc, Alfonso García-Ulloa, Iva M. Tolić, Stephen E. Kearsey

**Affiliations:** 1 Department of Zoology, University of Oxford, Oxford, United Kingdom; 2 Max Planck Institute of Molecular Cell Biology and Genetics, Dresden, Germany; 3 Division of Molecular Biology, Ruđer Bošković Institute, Zagreb, Croatia; Universita' di Milano, Italy

## Abstract

Analysis of accumulation of repair and checkpoint proteins at repair sites in yeast nuclei has conventionally used chemical agents, ionizing radiation or induction of endonucleases to inflict localized damage. In addition to these methods, similar studies in mammalian cells have used laser irradiation, which has the advantage that damage is inflicted at a specific nuclear region and at a precise time, and this allows accurate kinetic analysis of protein accumulation at DNA damage sites. We show here that it is feasible to use short pulses of near-infrared laser irradiation to inflict DNA damage in subnuclear regions of yeast nuclei by multiphoton absorption. In conjunction with use of fluorescently-tagged proteins, this allows quantitative analysis of protein accumulation at damage sites within seconds of damage induction. PCNA accumulated at damage sites rapidly, such that maximum accumulation was seen approximately 50 s after damage, then levels declined linearly over 200–1000 s after irradiation. RPA accumulated with slower kinetics such that hardly any accumulation was detected within 60 s of irradiation, and levels subsequently increased linearly over the next 900 s, after which levels were approximately constant (up to ca. 2700 s) at the damage site. This approach complements existing methodologies to allow analysis of key damage sensors and chromatin modification changes occurring within seconds of damage inception.

## Introduction

Single cell analysis of the localization of repair and checkpoint proteins has been of considerable use in both yeasts and mammalian cells for determining the temporal order and dependencies of protein accumulation at sites of DNA damage, thus helping to establish the order in which proteins function in repair and checkpoint pathways [1]–[3]. Protein localization can be correlated with cell cycle stage, showing that S phase entry or G1/G2 phase differences may affect repair pathway choices, and effects on cell cycle progression can be monitored. A number of methods have been used to effect DNA damage in yeasts. Double strand breaks can be generated by expression of site-specific endonucleases such as HO [4], [5] and I-SceI [6], exposure to chemical agents such as bleomycin-family antibiotics [7] or 4-NQO [8], or using γ or UV-C irradiation [9]. A wide range of chemical agents can effect nucleotide damage, such as the DNA methylating agent MMS, which generates lesions including N3-methyl adenine that cannot be bypassed by replicative DNA polymerases [10], potentially leading to fork stalling and collapse. Replication stress is commonly induced using hydroxyurea which, as a ribonucleotide reductase inhibitor reduces dNTP levels, leading to fork stalling. Replication fork stalling can also be induced using a polar replication fork barrier sequence such as *RTS1*, which can be activated by switching on the expression of a protein required for *RTS1* function [11]. These methods can be combined with strains expressing fluorescent fusion proteins to allow real-time analysis of repair processes but suffer a limitation in that there is a delay between induction of damage and analysis of the cellular response, making it difficult to study early (<60 s) events. An alternative method for DNA damage induction that has been widely used in mammalian cells is to use laser irradiation, which potentially allows repair responses to be visualized within seconds of damage induction ([12]). This approach initially used UVA lasers in conjunction with pre-sensitized DNA [13] and subsequently has been applied using longer wavelength lasers (e.g. [12], [14]–[16]). However this method has not been used in yeasts owing to technical problems associated with irradiation of a smaller nuclear volume.

We have previously used and characterised two-photon and single-photon ablation of intracellular structures in fission yeast such as microtubules and mitotic spindles [17]–[24]. We show here that it is feasible to use near-infrared (NIR) pulsed lasers for the analysis of DNA damage in yeasts. The NIR wavelength used (745 nm) is non-destructive and causes negligible heating [25], but irradiation with ultrashort low energy pulses (140 fs, 12–36 fJ) can effect three photon absorption with consequent DNA damage only in a central focussed region (ca. 300 nm diameter), which is considerably smaller than the diameter of yeast nuclei (1–2 µm). The NIR laser beam and the confocal imaging laser are parfocal at the region where multi-photon absorption occurs, allowing real time data acquisition. This approach complements existing techniques, and has significant advantages particularly for analysis of events occurring within seconds of DNA damage.

## Material and Methods

### Strains used and sample preparation

*Schizosaccharomyces pombe* strains used in this work are listed in Table S1. Cells were grown overnight (14–16 hours) on Yeast Extract Media agar plates with supplements: adenine, leucine, uracil, histidine and arginine (YE5S) at 25°C. For manipulation and imaging, fresh cells were resuspended in liquid Edinburgh Minimal Medium (EMM2) supplemented with adenine, leucine, uracil, histidine and arginine and transferred to the base of a 35 mm petri dish (MatTeck Corporation), the central region of which was coated with 2 µl of 2 mg/ml lectin (L2380, Sigma-Aldrich). Free cells were removed by washing with EMM2, the petri dish was filled with 300 µl of EMM2, covered with a coverslip and sealed with silicone (GE Bayer Silicones). During the manipulation and imaging of cells on the microscope, the sample was kept at 25°C in a chamber. For analysis of bleocin-treated cells by fluorescence microscopy, cells were washed in water and mounted in 1.2% low melting temperature agarose. Images were collected using a Zeiss Axioplan microscope, coupled to a Hamamatsu ORCA ER camera; open source MicroManager software [26] was used to control the camera and microscope.

### Laser scanning confocal microscopy and laser irradiation

Live-cell images were taken using an upright microscope, with infinity-corrected optics (Zeiss LSM 780 NLO). For GFP excitation we used the 488 nm line of a multi-line Argon-Ion laser (LASOS), a 488 nm notch filter and a Plan-Apochromat 63x/1.40 Oil DIC objective (Zeiss); the set-up is summarised in Figure S1. Emission was detected in the range of 490–580 nm. During laser irradiation no images were taken. Before and after DNA damage, time-lapse z-stacks of 8–11 optical sections with a 500 nm z-distance, were taken at 3 or 30 s time interval using unidirectional scanning mode at 3.38 µs scanning speed. All images have a *xy*-pixel size of 225 nm.

DNA damage was performed using a two-photon Ti:Sapphire femtosecond pulsed laser (Chameleon Vision II, Coherent), tuned to a wavelength of 745 nm and a theoretical pulse duration of 140 femtoseconds at 80 MHz. The beam was coupled to the bleaching port of the Zeiss LSM 780 NLO laser scanning microscope. The light path of the pulsed laser was different from the path of the imaging one. The pulsed laser light was reflected onto the objective by a short pass dichroic mirror SP690. The laser power before the objective was 857 mW, measured at the maximum output power of 2480 mW using a power meter (Coherent). The power at the sample corresponding to the range 12%–30% of maximum output power was ∼12–32 mW, estimated from the objective transmission at 745 nm (12%). The irradiations were performed on a user-defined region of interest (ROI) of 0.775 µm×0.225 µm, drawn inside of the nucleus before starting acquisition. The irradiation was achieved by scanning the laser at the power mentioned above over the ROI, for 40 µs total exposure time.

### Quantification and image analysis

**Figure 1:**
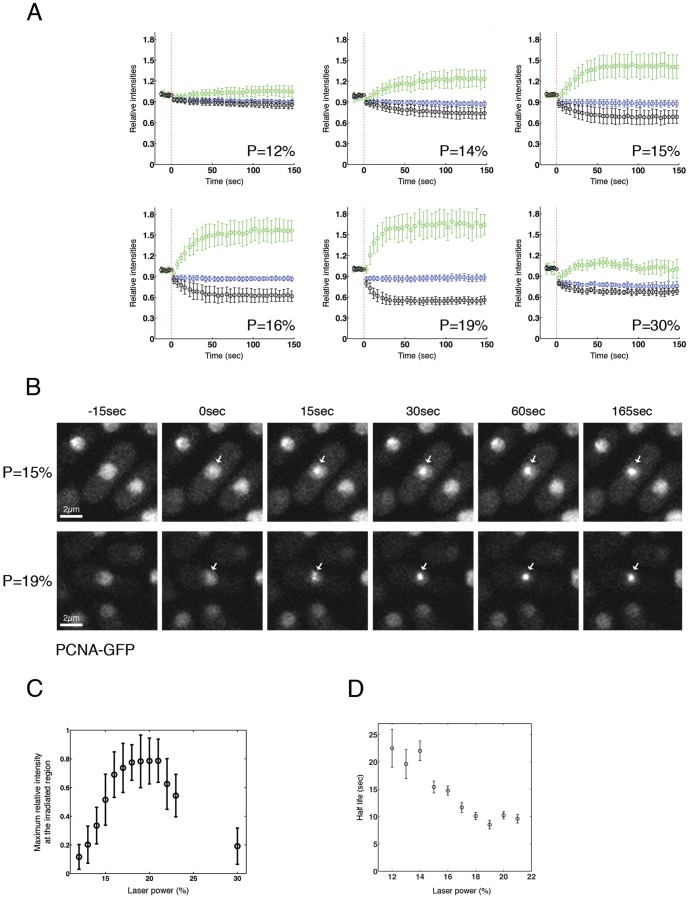
PCNA is recruited at the site of DNA damage in a dose-dependent manner. (**A**) GFP-PCNA expressing cells were subjected to micro-irradiation with indicated powers (P) and live series of images over a time period of 150 seconds were recorded. The average intensity of GFP-PCNA in the entire nucleus (blue), at the irradiated site (green) and in the area of the nucleus that was not irradiated (black) was quantified as described in Materials and Methods. Between 10 and 20 cells were processed for every power; error bars show standard deviation. The relationship between laser power and energy delivered is shown in Figure S3A. (**B**) GFP-PCNA expressing cells were exposed to laser micro-irradiation with powers shown and images were acquired at the indicated times. Arrows indicate sites of irradiation. (**C**) Total amount of protein accumulation for every power was calculated as described in Materials and Methods (**D**) Half life of PCNA accumulation values for every power, calculated as described in Materials and Methods.

**Figure 2:**
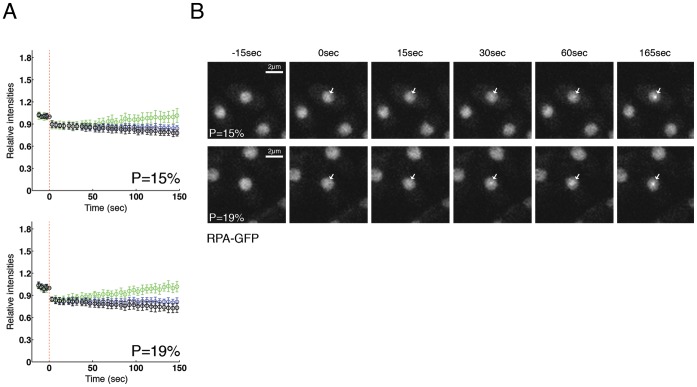
RPA is recruited in sites of DNA damage after laser irradiation. (**A**) Cells expressing Rad11-GFP (RPA subunit) were micro-irradiated with powers 15% and 19%, and fluorescence was quantified and plotted as indicated in Figure 1A. Between 10 and 15 cells per power were analyzed. (**B**) Two examples of RPA-GFP micro-irradiated cells from Figure 2A are shown. Arrows indicate irradiated regions.

**Figure 3:**
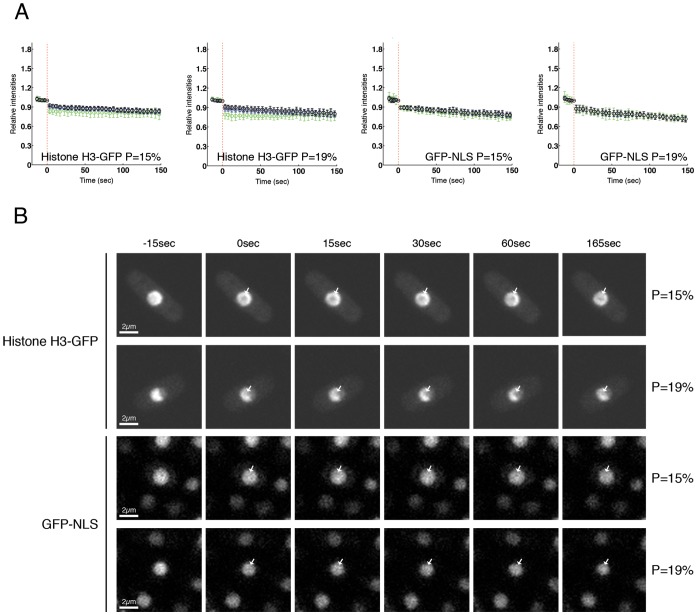
Histone H3 and GFP-NLS are not accumulated in the damage site of the ablated nucleus. (**A**) Cells expressing histone H3-GFP or GFP-NLS were irradiated with powers of 15% and 19%, and intensity of fluorescence was measured and plotted as in Figure 1A. Between 10 and 15 cells per power and per strain were analyzed. (**B**) Histone H3-GFP (upper panels) or GFP-NLS (lower panels) expressing cells were laser-irradiated with indicated powers and images from indicated time points are shown. Arrows show sites of irradiation.

To analyze accumulation of proteins at damage sites in the cell nucleus, the z-stacks were sum projected using ImageJ 1.47c (National Institutes of Health, USA). For quantifications described in Figures 1A, 2A, 3A and S3, sum intensities were read out using the regular selection tools already existing in ImageJ, circle for the nucleus and 3×3 pixels square for damage sites. Fluorescence intensity of the non-irradiated part of the nucleus was calculated by subtracting the intensity of the irradiated region from the total intensity within the nucleus. For all ROIs the intensity values were normalized for each time point to the intensity value at first frame before irradiation.

**Figure 4:**
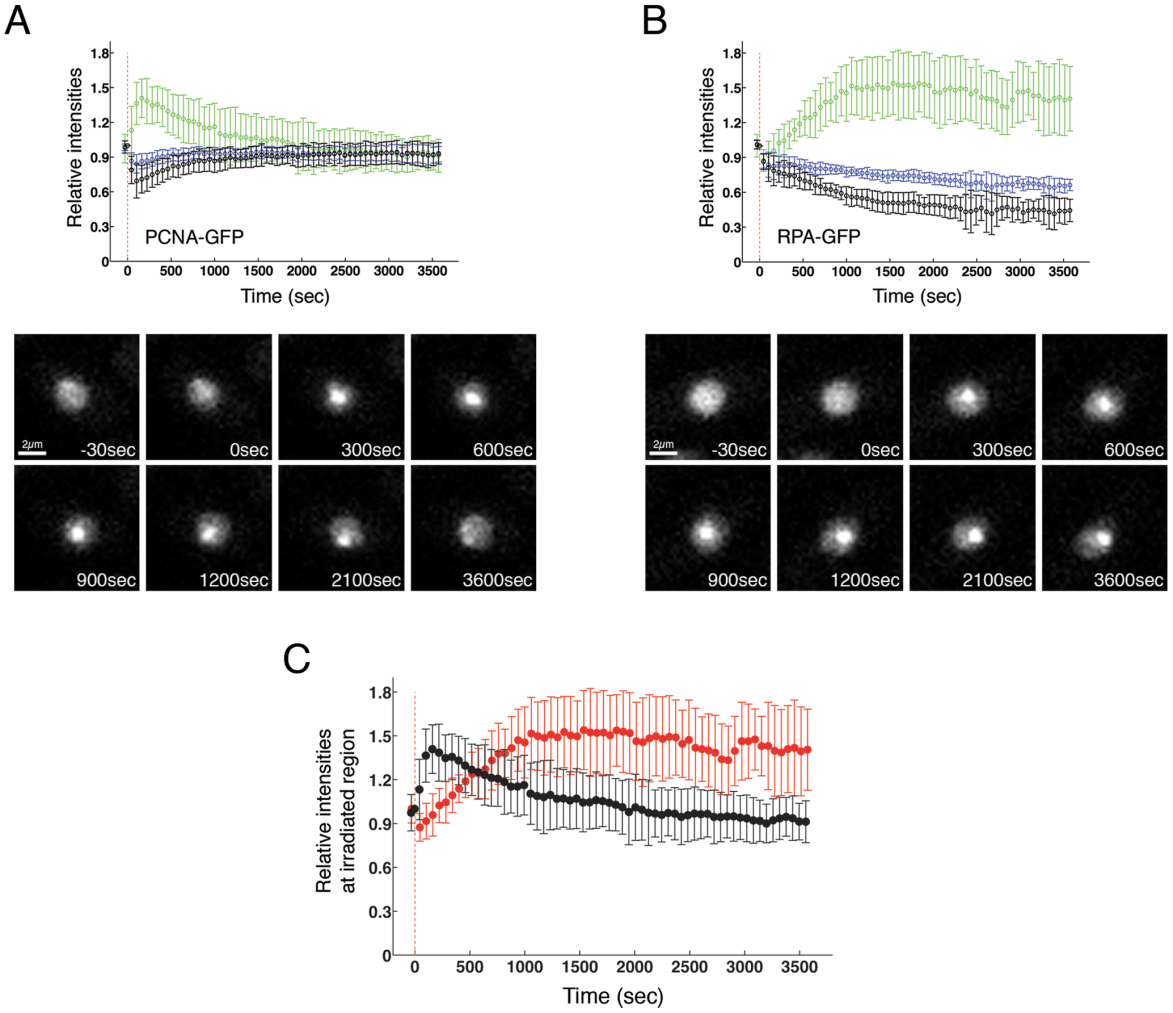
PCNA is recruited to the damage site before RPA. GFP-PCNA (A) or RPA-GFP (B) expressing cells were irradiated at a power of 15% and cells were monitored every 30 seconds over 60 minutes. The average intensity of GFP-PCNA at the irradiated site (green), in the entire nucleus (blue) and in the area of the nucleus that was not irradiated (black) was quantified and plotted as indicated in Materials and Methods. 10–15 cells per strain were irradiated (upper panels). Example images of both strains are shown in the lower panels (**C**) The intensity of the fluorescence at the site of the damage was quantified and plotted for both strains in the same graph to allow easier comparison of the kinetics. Black: GFP-PCNA; Red: RPA-GFP

For the quantifications in Figures 4A-C, we took into consideration movies acquired for one hour. In this situation, the nucleus is moving, making it difficult to use the regular selection tools from ImageJ. A custom-made ImageJ plugin (see Script S1) was used to perform the analysis, which consists of two modules, one for quantifying the accumulation of proteins in the ablation region and the other to quantify the signal in the nucleus. The irradiated region was analyzed by fitting a circle of 3 pixels radius centred at the most intense region of the nucleus over the duration of the experiment. The sum of pixel intensity values in the irradiated region was obtained as a function of time. The cell nucleus was analyzed by detecting the region containing the nucleus over the length of the experiment.

The total relative amount of protein accumulation and the half-life of accumulation (time needed to accumulate 50% of the maximum amount) were obtained by fitting the measured values with the following exponential equation:
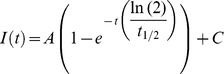
where *I(t)* is the normalized intensity as function of time, *A* is the total amount of protein accumulation, *t_1/2_* is the half-life and *C* is the new normalized level of fluorescence immediately after laser irradiation.

## Results and Discussion

### PCNA and RPA are detected at sites of DNA damage after NIR laser irradiation of fission yeast nuclei in a dose-dependent manner

The use of NIR laser irradiation (745 nm) for induction of DNA damage relies on multiphoton absorption which only takes place at a highly focussed femtolitre volume of the laser beam. This effectively delivers UV photon quanta without the requirement for DNA presensitization, or problems of UV absorption by microscope components or biological material. This irradiation is predicted to generate a variety of types of DNA damage, including UV photoproducts such as CPDs [27], and double-strand breaks (DSBs) [28]. For initial experiments we chose to analyse GFP-tagged PCNA (proliferating cell nuclear antigen), which is a central protein in repair and replication and participates in a number of repair pathways [29]–[32]. PCNA encircles DNA as a trimer, forming a sliding clamp that tethers a wide variety of proteins such as polymerases to DNA [33] and several studies using mammalian cells have shown a recruitment of PCNA to DNA repair sites after local laser micro-irradiation of the nucleus [16], [34]–[38]. As previously reported [31], DSB generation in live fission yeast cells caused formation of GFP-PCNA foci after treating exponentially growing cells with bleocin (Figure S2).

We irradiated a specific region (775×225 nm) of the nucleus of cells expressing GFP-PCNA, while simultaneously imaging the cell every 3 s over 150 s. Initially, different irradiations were performed, varying the laser power in the sample plane in a range from 12% to 30% of total laser power (% power related to energy delivered in Figure S3A). GFP-PCNA fluorescence was quantified over the time course in the entire nucleus, at the irradiated site and in the region of the nucleus that was not irradiated (Figures 1A, 3B, Movies S1-13). After laser irradiation, photobleaching was observed proportional to the power used, resulting in a decreased GFP-PCNA fluorescence in the entire nucleus compared with the intensity before irradiation. With all powers used, an increase in GFP-PCNA fluorescence at the irradiated region was observed in seconds, with a corresponding decrease in fluorescence outside the irradiated region, while total fluorescence in the entire nucleus remained constant, indicating rapid PCNA recruitment to the damage site (Figure 1A-B). These results were consistent with similar experiments performed in human cells that also showed PCNA recruitment to sites of the damage within a few seconds after irradiation [16], [37].

When analysing the total relative amount of protein accumulation and the time needed for protein accumulation, we observed that PCNA accumulation at the irradiated region increased in a dose-dependent manner when cells were irradiated in the range of 12% to 18% (Figure 1C). When doses between 18% and 21% were used, levels of accumulation remained constant. Finally, the total recruitment of GFP-PCNA at the irradiated region started to decrease if doses over 21% were applied (Figure 1C), possibly as excessive cell damage has a detrimental effect on the response to DNA damage. The time needed for PCNA to accumulate to 50% of the maximum value decreased in the power range 12% to 19%, and was approximately constant in the range 19–21% (Figure 1D). This indicates that the rate of PCNA recruitment is dose-dependent up to a certain level of damage.

These results show that laser irradiation of *S. pombe* nuclei leads to a repair response that can be detected by monitoring the accumulation of fluorescently labelled PCNA. From these results we selected powers of 15% and 19% as most appropriated for following experiments, as 19% represented the lowest power that showed the maximum accumulation of PCNA, while 15% gave an accumulation around 60% of this value.

The heterotrimeric replication protein A (RPA) complex plays a similar role in replication and in repair, stabilizing regions of single-stranded DNA during resynthesis reactions and can be used as an indicator of DNA end processing for homologous recombination [39]–[41]. It also plays a key role in activation of checkpoint responses via ATR [42]. To compare the kinetics of RPA recruitment with PCNA, we performed laser irradiation experiments using a strain where the largest subunit of RPA is GFP tagged (Rad11-GFP) [43], with irradiation levels of 15% and 19%. As seen with PCNA, photobleaching was detected after irradiation (Figure 2A) and recruitment of RPA was observed from approximately 50 s after irradiation, although levels were continuing to rise at 150 s, unlike the situation with PCNA (Figure 2A-B, Movies S14, S15). As expected, RPA-GFP foci formation was also detected after treating cells with bleocin, as seen for PCNA (Figure S2).

The slower recruitment of RPA compared to PCNA may reflect the need for DNA end resection to generate ssDNA for RPA loading. This result agrees with the fact that PCNA can play other roles in some repair pathways, prior to its role as an auxiliary factor for polymerases. Some studies propose that PCNA could help to recruit essential proteins for nucleotide excision repair, such as XP-G and XP-A [44]–[47], or for base excision repair, such as glycosylase NEIL1 [48], AP endonucleases 1 and 2 or uracil-DNA glycosylase 2 (reviewed in [32]), and extensive DNA processing might not be required prior to accumulation. In addition, a recent study proposed PCNA as a new factor in the Exo1 resection pathway, promoting processive DNA end resection by Exo1 binding, apparently by assembling with DNA ends in an RFC-independent reaction [49]. Thus our results are consistent in showing that PCNA is recruited at a very early step and potentially could recruit other repair proteins prior to extensive generation of ssDNA.

### Histone H3 and NLS-GFP are not accumulated at damage sites of the ablated nucleus

Although core histones are modified after DNA damage to trigger DNA repair [50], [51], they are not accumulated at sites of damage. We could therefore use a strain where histone H3 is GFP-tagged to check whether accumulation of protein after irradiation was an artefact associated with laser ablation. Contrary to observations made with GFP-PCNA or RPA-GFP, GFP-tagged histone H3 was not accumulated at the irradiated region and only bleaching was detected (Figure 3A-B upper panels, Movies S16, S17). Most histone H3 is bound to DNA and this could impair nonspecific recruitment of H3 to the site of DNA damage. To test if a free nuclear protein with no DNA binding properties could potentially bind damaged DNA nonspecifically following laser irradiation, we also tested how GFP, tagged with a nuclear localization sequence (NLS), was affected. Results similar to those with histone H3 were obtained, with only bleaching of the irradiated region during the time course (Figure 3A-B lower panels, Movies S18, S19). This indicates that movements of PCNA and RPA to the damage site are specific for those two proteins and proteins are not recruited nonspecifically.

### RPA remains at the damage site after PCNA is delocalized

We examined the kinetics of PCNA and RPA accumulation at the site of damage over a longer time course of 60 min. As previously shown in Figure 1, PCNA was very rapidly recruited at the damage site after irradiation, and only 10–25 s were required for PCNA to be recruited to 50% of its maximum value depending on the power used (Figure 1D). Approximately 200 seconds after irradiation, GFP-PCNA started to decrease at the ablated region, and levels declined linearly with time over the next ca. 15 min, while levels of PCNA in the rest of the nucleus recovered (Figure 4A, Movie S20). This suggests that PCNA is released from the damage site a few minutes after irradiation, perhaps reflecting an initial role in DNA repair very soon after damage is generated. Conversely, RPA was recruited at the damage site more slowly and around 500 s were required for the protein to be recruited to 50% of its maximum level, reaching a constant level around 1000 s after the damage was induced. No reduction in RPA-GFP fluorescence at the irradiated region was observed during the subsequent 45 min (Figure 4B, Movie S21), consistent with previous results using ionizing radiation [52]. RPA recruitment on chromatin is almost at a maximum when PCNA is largely released from the irradiated area (approximately 15 min after irradiation, Figure 4C). These results fit a model where PCNA is involved in early steps of DNA repair, and perhaps is needed for generation of ssDNA, prior to its role at later time points during the repair process as a polymerase loader [29], [32], [34], [49], [53]. Moreover, our results support the recent finding that PCNA is loaded onto DSBs as an early event in repair pathways [49].

In spite of intensive study of the localization of repair and checkpoint factors to damage foci in yeasts, technical problems with conventional damage-induction procedures make it difficult to analyse this process on a time scale of seconds after damage has occurred. The results described here indicate that such real-time monitoring of repair factors after NIR laser irradiation is feasible in fission yeast. Such monitoring is important to establish the role of early damage events, such as the role of mammalian KDM4D histone methylase in promoting DSB repair [54], and the recruitment of FUS at DNA sites in neurons [55]. Single cell irradiation also allows damage responses to be correlated with cell cycle stage and stochastic cell-cell differences to be explored. One issue with NIR laser irradiation and laser irradiation in general, is that a single type of DNA damage is not generated but this may be addressed by using longer wavelength irradiation (1050 nm), which leads to preferential formation of DSBs [25], [56].

## Supporting Information

Figure S1 **Confocal set up showing arrangement of NIR irradiation laser with respect to confocal imaging laser.** NF488, notch filter reflects the 488 nm laser, but lets the rest of the visible spectrum pass; SP690, short pass dichroic mirror, lets wavelengths below 690 nm pass; M1 and M2, scanning mirrors.
(TIF)

Figure S2 **PCNA and RPA form nuclear foci after DNA damage generation.** PCNA-GFP, RPA-GFP, Histone H3-GFP and GFP-NLS expressing cells were grown in Yeast Extract Media with adenine, leucine, uracil supplements (YE3S) at 30°C until mid-log phase and then were treated with bleocin 0.5 µg/ml for 30 min before imaging. Arrows show examples of foci.
(TIF)

Figure S3 (A) Relationship between % laser power and energy delivered. (B) PCNA is recruited to the site of DNA damage in a dose-dependent manner. PCNA-GFP expressing cells were micro-irradiated with indicated powers (P) and fluorescence was quantified and plotted as in Figure 1A. Between 10 and 15 cells were processed for every power. Error bars show standard deviation.
(TIF)

Table S1 **Strains used in this work.**
(PDF)

Script S1 **Script used for analysis of protein accumulation for long time courses.**
(PDF)

Movie S1 Cells expressing GFP-tagged proteins were micro-irradiated as described in Materials and Methods with the indicated power; images were recorded every 3 seconds over a time period of 150 seconds for movies 1–19 and every 30 seconds over 60 minutes for movies 20 and 21. This is PCNA-GFP, P = 12%.
(M4V)

Movie S2 **PCNA-GFP, P = 13%.**
(M4V)

Movie S3 **PCNA-GFP, P = 14%.**
(M4V)

Movie S4 **PCNA-GFP, P = 15%.**
(M4V)

Movie S5 **PCNA-GFP, P = 16%.**
(M4V)

Movie S6 **PCNA-GFP, P = 17%.**
(M4V)

Movie S7 **PCNA-GFP, P = 18%.**
(M4V)

Movie S8 **PCNA-GFP, P = 19%.**
(M4V)

Movie S9 **PCNA-GFP, P = 20%.**
(M4V)

Movie S10 **PCNA-GFP, P = 21%.**
(M4V)

Movie S11 **PCNA-GFP, P = 22%.**
(M4V)

Movie S12 **PCNA-GFP, P = 23%.**
(M4V)

Movie S13 **PCNA-GFP, P = 30%**
(M4V)

Movie S14 **PRPA-GFP, P = 15%.**
(M4V)

Movie S15 **RPA-GFP, P = 19%.**
(M4V)

Movie S16 **H3-GFP, P = 15%.**
(M4V)

Movie S17 **H3-GFP, P = 19%.**
(M4V)

Movie S18 **GFP-NLS, P = 15%.**
(M4V)

Movie S19 **GFP-NLS, P = 19%.**
(M4V)

Movie S20 **PCNA-GFP, P = 15%.**
(M4V)

Movie S21 **RPA-GFP, P = 15%.**
(M4V)
