# Supplementary figures and images for: Real-Time Imaging of DNA Damage in Yeast Cells Using Ultra-Short Near-Infrared Pulsed Laser Irradiation
Source: PLoS One. 2014 Nov 19;9(11):e113325. doi: 10.1371/journal.pone.0113325 (PMC4237433; doi:10.1371/journal.pone.0113325)

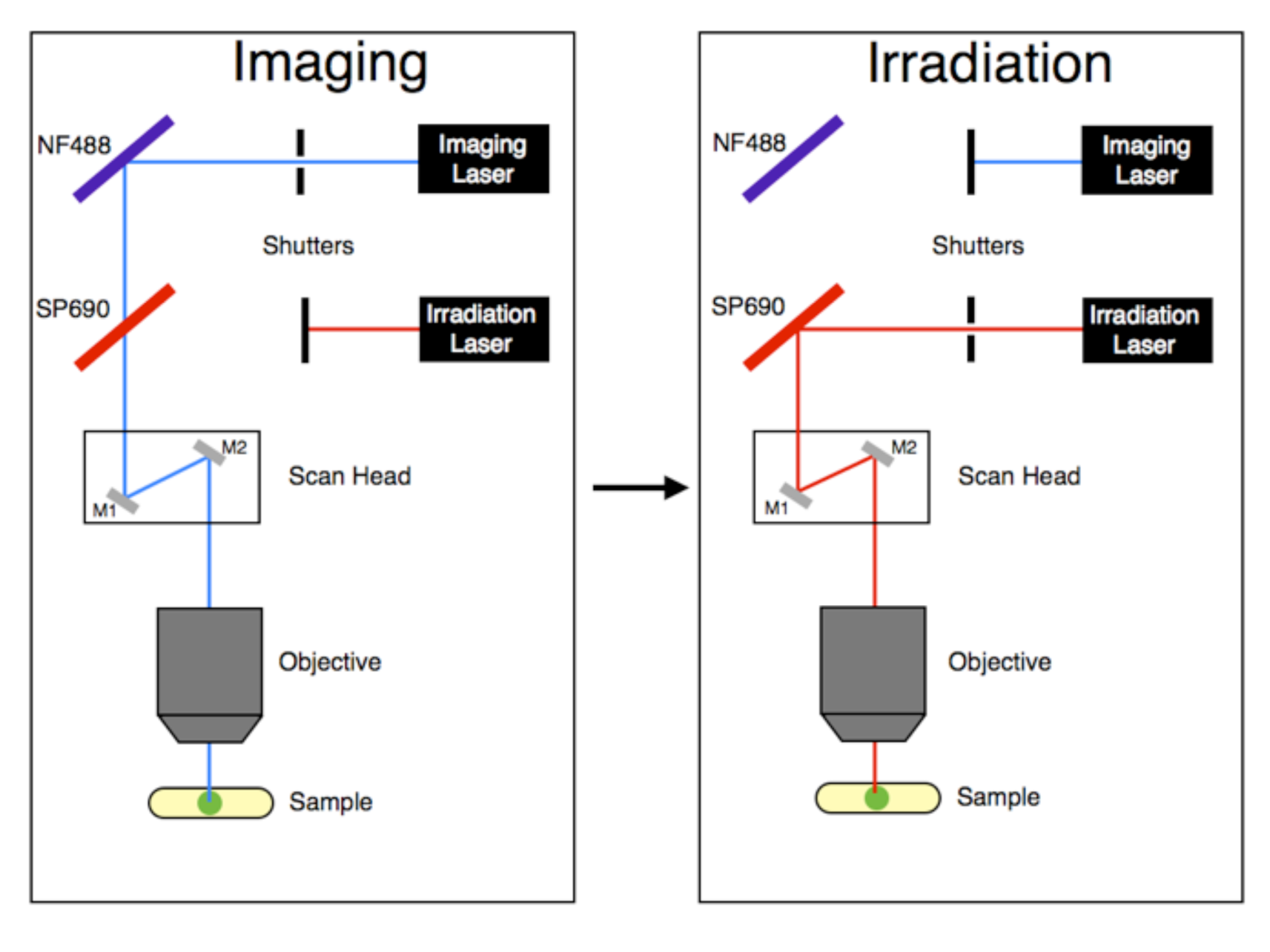

Supplement: Figure S1 — Confocal set up showing arrangement of NIR irradiation laser with respect to confocal imaging laser. NF488, notch filter reflects the 488 nm laser, but lets the rest of the visible spectrum pass; SP690, short pass dichroic mirror, lets wavelengths below 690 nm pass; M1 and M2, scanning mirrors. (TIF) [file pone.0113325.s001.tif]

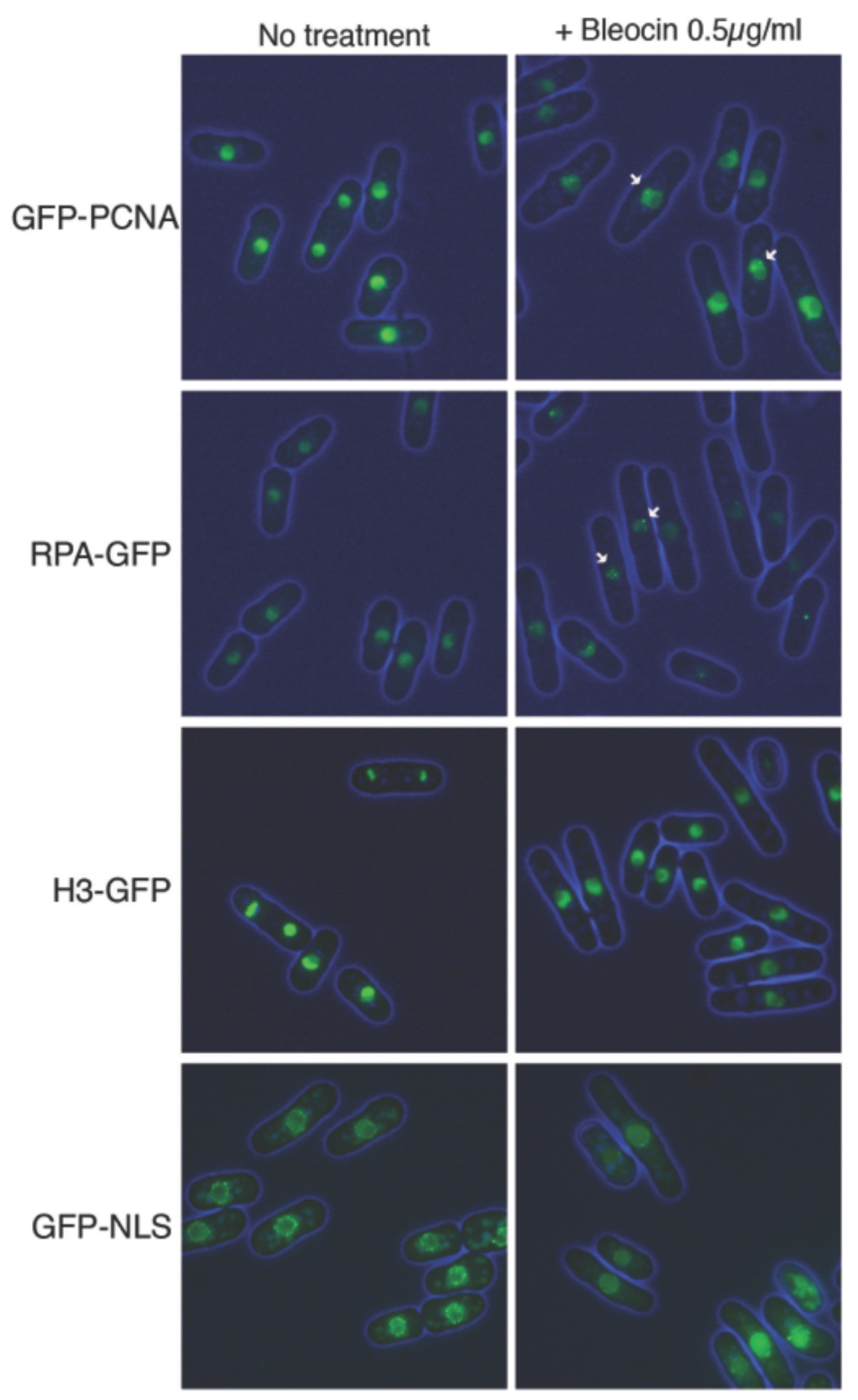

Supplement: Figure S2 — PCNA and RPA form nuclear foci after DNA damage generation. PCNA-GFP, RPA-GFP, Histone H3-GFP and GFP-NLS expressing cells were grown in Yeast Extract Media with adenine, leucine, uracil supplements (YE3S) at 30°C until mid-log phase and then were treated with bleocin 0.5 µg/ml for 30 min before imaging. Arrows show examples of foci. (TIF) [file pone.0113325.s002.tif]

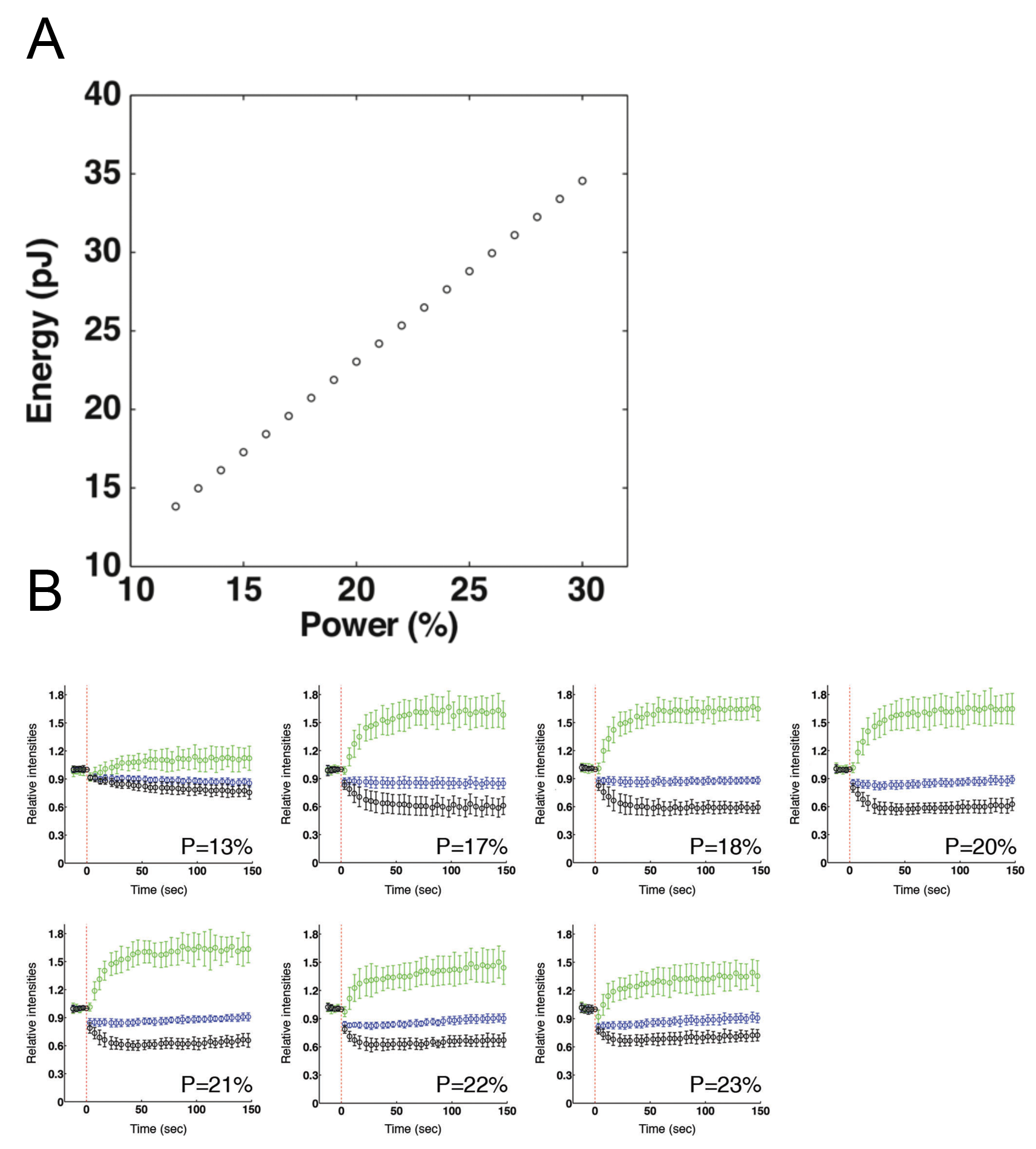

Supplement: Figure S3 — (A) Relationship between % laser power and energy delivered. (B) PCNA is recruited to the site of DNA damage in a dose-dependent manner. PCNA-GFP expressing cells were micro-irradiated with indicated powers (P) and fluorescence was quantified and plotted as in Figure 1A. Between 10 and 15 cells were processed for every power. Error bars show standard deviation. (TIF) [file pone.0113325.s003.tif]
