# Supplementary material for: Real-Time Imaging of DNA Damage in Yeast Cells Using Ultra-Short Near-Infrared Pulsed Laser Irradiation
Source: PLoS One. 2014 Nov 19;9(11):e113325. doi: 10.1371/journal.pone.0113325 (PMC4237433; doi:10.1371/journal.pone.0113325)
Supplement: Table S1 — Strains used in this work. (PDF) [file pone.0113325.s004.pdf]

Table S1. Strains used in this work.

Yeast strains used	
1304	*h+ leu1-32 ura4::pcn1-GFP*
1821	*h- leu1-32 ura4-D18 hht2-GFP::ura4*
1992	*h+ ade6-216 leu1-32 rad11-GFP::kanR*
3257	*h+ dis1prom-GFP-lacI-NLS::his7+*
