# Supplementary material for: Real-Time Imaging of DNA Damage in Yeast Cells Using Ultra-Short Near-Infrared Pulsed Laser Irradiation
Source: PLoS One. 2014 Nov 19;9(11):e113325. doi: 10.1371/journal.pone.0113325 (PMC4237433; doi:10.1371/journal.pone.0113325)
Supplement: Script S1 — Script used for analysis of protein accumulation for long time courses. (PDF) [file pone.0113325.s005.pdf]

```
# Long_movies_plugin author Alfonso Garcia Ulloa
# Real-time imaging of DNA damage in yeast cells using ultra-short
# near-infrared pulsed laser irradiation
# Estrella Guarino, Gheorghe Cojoc, Alfonso García Ulloa,
# Iva M. Tolić and Stephen E. Kearsey
# compiled code available from
# https://dl.dropboxusercontent.com/u/606434/plugins/Long_movies_plugin.py

# Loading neccesary libraries
from java.lang import InterruptedException, Runnable, Thread
from java.beans import PropertyChangeListener
from java.util import Random
from java.util.concurrent import ExecutionException
from javax.swing import SwingWorker, SwingUtilities
from javax.swing import BorderFactory
from java.awt import Toolkit as awtToolkit
from java.awt import BorderLayout as awtBorderLayout
from java.awt import Cursor as awtCursor
from java.awt import Insets as awtInsets
from javax.swing import JLabel,JFileChooser, JProgressBar, JPanel, JButton,
JFrame, JDesktopPane,JTextField, SwingConstants, JCheckBox
from java.awt import Component, GridLayout
from java.awt.event import MouseAdapter
import math,os,sys,copy
from ij.measure import CurveFitter
from ij.io import FileSaver
from math import atan2,degrees
from ij.plugin import Converter
from ij.plugin import ZProjector as ZP
from ij.plugin import Slicer as Sl
from ij.plugin.filter import Binary,RankFilters,GaussianBlur
from ij.plugin import ChannelSplitter as CS
from ij.gui import Roi,ProfilePlot,ShapeRoi
from java.awt import Color, Dimension
from java.io import File
from time import sleep
import timeit
from java.awt.geom import Ellipse2D

#######################################################################
#Swing Workers, all the action happens in the background
#######################################################################
class Load(SwingWorker):

      def __init__(self, gui):
            self.gui = gui
            SwingWorker.__init__(self)

      def doInBackground(self):



            od = OpenDialog("Choose a file", None)
            global filename
            filename = od.getFileName()
```

```
            if filename is None:
                  print "User canceled the dialog!"
            else:
                  global directory
                  directory = od.getDirectory()
            filepath = directory + filename
            print "Selected file path:", filepath
            global imp1,imp2,plotprof
            IJ.run("Bio-Formats Importer", "open=["+filepath+"] autoscale
color_mode=Colorized view=Hyperstack stack_order=XYCZT")
            IJ.run("Green Fire Blue")
            imp1= WindowManager.getCurrentImage()
            imp2=imp1.duplicate()
            if imp1.getBitDepth()!=8:
                  StackConverter(imp1).convertToGray8()
            IJ.setTool('line')
            IJ.makeLine(0,0,1,1)
            IJ.run('Plot Profile')
            plotprof=WindowManager.getCurrentImage()
            print imp1.getID()
            IJ.selectWindow(imp1.getID())

      def done(self):
            try:
                self.get()  #raise exception if abnormal completion
                awtToolkit.getDefaultToolkit().beep()

                self.gui.loadButton.enabled = True
                self.gui.cursor = None  #turn off the wait cursor
            except ExecutionException, e:
                awtToolkit.getDefaultToolkit().beep()

                self.gui.loadButton.enabled = True
                self.gui.cursor = None  #turn off the wait cursor
                raise e.getCause()

###########################################################################
class Task(SwingWorker):

      def __init__(self, gui):
            self.gui = gui
            SwingWorker.__init__(self)

      def doInBackground(self):
            plotprof.close()
            treshold = int(self.gui.tresholdField.text)
            Hm= int(self.gui.HmField.text)
            Hr= int(self.gui.HrField.text)
            tol= int(self.gui.tolField.text)
            tic=timeit.default_timer()
            ip1=imp1.getProcessor()
            Width=imp1.getWidth()
            Height=imp1.getHeight()
            imp1.setSlice(1)
            A=ip1.getIntArray()
```

```
        def IsHoodMin(A,x,y,W,H,Tol):
            if not (x-Tol<0 or x+Tol+1>W or y-Tol<0 or y+Tol+1>H):
                minV=A[x][y]
                for i in range(x-Tol,x+Tol+1):
                    for j in range(y-Tol,y+Tol+1):
                        minV=min(minV,A[i][j])
                if minV==A[x][y]:
                    return True
                else:
                    return False

            else:
                return 'error',x,y,x-1<0,x+2>W ,y-1<0,y+2>H
        def HoodMean(A,x,y,W,H,HoodRadius):
            if not (x-HoodRadius<0 or x+HoodRadius+1>W or y-HoodRadius<0
or y+HoodRadius+1>H):
                mean=0
                for i in range(x-HoodRadius,x+1+HoodRadius):
                    for j in range(y-HoodRadius,y+1+HoodRadius):
                        mean=mean+A[i][j]
                return mean/9.0
            else:
                return 0

        def HoodMin(A,x,y,W,H,HoodRadius,ip):
            if not (x-HoodRadius<0 or x+HoodRadius+1>W or y-HoodRadius<0
or y+HoodRadius+1>H):
                minV=A[x][y]
                for i in range(x-HoodRadius,x+HoodRadius+1):
                    for j in range(y-HoodRadius,y+HoodRadius+1):
                        minV=min(minV,A[i][j])
                for i in range(x-HoodRadius,x+HoodRadius+1):
                    for j in range(y-HoodRadius,y+HoodRadius+1):
                        if minV+1>A[i][j] and minV-1<A[i][j] and
A[i][j]>27 and A[i][j]<255:
                            ip.setColor(255)
                            ip.drawPixel(i,j)
        def HoodMax(A,x,y,W,H,tr,HoodRadius,Hmt,tolerance,ip,ip2):
            if not (x-HoodRadius<0 or x+HoodRadius+1>W or y-HoodRadius<0
or y+HoodRadius+1>H):
                maxV=A[x][y]
                for i in range(x-HoodRadius,x+HoodRadius+1):
                    for j in range(y-HoodRadius,y+HoodRadius+1):
                        maxV=max(maxV,A[i][j])
                for i in range(x-HoodRadius,x+HoodRadius+1):
                    for j in range(y-HoodRadius,y+HoodRadius+1):
                        if maxV+tolerance>A[i][j] and maxV-
tolerance<A[i][j] and A[i][j]>tr and A[i][j]<255 and
HoodMean(A,i,j,W,H,HoodRadius)>Hmt:
                            ip1.setColor(255)
                            ip1.drawPixel(i,j)

            else:
                return 'error',x,y,x-1<0,x+2>W ,y-1<0,y+2>H
        #print treshold,Width,Height,imp1.getStackSize()
```

```
            ResultLayer=imp2

            RLip=ResultLayer.getProcessor()

            #print ResultLayer,RLip

            for slice in range(1,imp1.getStackSize()+1):
                  #print slice
                  imp1.setSlice(slice)
                  ResultLayer.setSlice(slice)
                  A=ip1.getIntArray()
                  toc=timeit.default_timer()
                  #print toc
                  IJ.showStatus(str(slice)+'/'+str(imp1.getStackSize())+'
Elapsed time: '+str((toc - tic)/60.0))
                  IJ.showProgress(slice, imp1.getStackSize())
                  for i in range(1,Width-2):
                        for j in range(1,Height-2):

      HoodMax(A,i,j,Width,Height,treshold,Hr,Hm,tol,ip1,RLip)
                  RLip.setThreshold(200, 255, ImageProcessor.NO_LUT_UPDATE)
                  #ResultLayer.show()
                  self.super__setProgress(int(slice/imp1.getStackSize()*100))
            toc=timeit.default_timer()
            print (toc - tic)/60.0 #elapsed time in seconds
            IJ.showStatus('FIN')
            #FileSaver(ResultLayer).saveAsTiffStack(directory+filename[:-
4]+'Track'+filename[-4:])
            FileSaver(imp1).saveAsTiffStack(directory+filename[:-
4]+'Track'+filename[-4:])
            #ResultLayer.show()
            cal=imp1.getCalibration()
            def doSomething(imp,loc):
                  """ A function to react to a mouse click on an image canvas.
"""
                  #print "clicked on: " ,imp
                  #print loc.getX(),loc.getY()
                  #print cal.getX(loc.getX()),cal.getX(loc.getY())
                  self.gui.xtrackField.text=str(cal.getX(loc.getX()))
                  self.gui.ytrackField.text=str(cal.getX(loc.getY()))
                  ip=imp.getProcessor()



                  if self.gui.checkbox.isSelected():
                        if ip.getPixel(int(loc.getX()),int(loc.getY()))>250:
                              ip.setColor(0)
                              ip.drawPixel(int(loc.getX()),int(loc.getY()))

      FileSaver(imp).saveAsTiffStack(directory+filename[:-4]+'Track'+filename[-
4:])
                        else:
                              ip.setColor(255)
                              ip.drawPixel(int(loc.getX()),int(loc.getY()))
```

```
      FileSaver(imp).saveAsTiffStack(directory+filename[:-4]+'Track'+filename[-
4:])
            class ML(MouseAdapter):
                  def mousePressed(self, event):
                        canvas = event.getSource()
                        imp = canvas.getImage()
                        loc= canvas.getCursorLoc()
                        doSomething(imp,loc)

            listener = ML()

            #for imp in map(WindowManager.getImage, WindowManager.getIDList()):
            win = imp1.getWindow()
            win.getCanvas().addMouseListener(listener)
            IJ.setTool("oval")
            '''
            table1 = ResultsTable()
            roiM = RoiManager(True)
            pa = ParticleAnalyzer(ParticleAnalyzer.ADD_TO_MANAGER,
Measurements.AREA | Measurements.CENTROID| Measurements.ELLIPSE|
Measurements.CIRCULARITY| Measurements.SLICE| Measurements.RECT, table1, 3,
Double.POSITIVE_INFINITY, 0.0, 1.0)
            pa.setHideOutputImage(True)
            pa.setRoiManager(roiM)
            for i in range(imp1.getStackSize()):
                  imp1.setSlice(i+1)
                  if pa.analyze(imp1):
                        print "All ok"
                  else:
                        print "There was a problem in analyzing"
            a = table1.getColumn(0)
            frame = table1.getColumn(27)
            X = table1.getColumn(6)
            Y = table1.getColumn(7)
            Major = table1.getColumn(15)
            Minor = table1.getColumn(16)
            bx = table1.getColumn(11)
            by = table1.getColumn(12)
            angle = table1.getColumn(31)
            Circ = table1.getColumn(18)
            w= table1.getColumn(13)
            h = table1.getColumn(14)
            #print a,b
            print filename

            means = []
            for roi in roiM.getRoisAsArray():
                  imp2.setRoi(roi)
                  stats = imp2.getStatistics(Measurements.MEAN)
                  means.append(stats.mean)
            print len(means)

            name=filename[:-4]+'_K.txt'
            filepath1 = str(od.getDirectory())+name
```

```
            print filepath1,name
            o = open(filepath1,"wb") #open for write
            line='%header SPB1 Track
\n%i\tA\tMeanGrayValue\tX\tY\tBx\tBy\tMajor\tWidth\tCircularity\n'
            o.write(line)

            for i in range(len(a)):

      line=str(int(frame[i]))+'\t'+str(a[i])+'\t'+str(means[i])+'\t'+str(X[i])+'
\t'+str(Y[i])+'\t'+str(bx[i])+'\t'+str(by[i])+'\t'+str(Major[i])+'\t'+str(w[i])+
'\t'+str(Circ[i])+'\n'
                  o.write(line)

            o.write(str(i))
            o.close()
            '''


      def done(self):
            try:
                self.get()  #raise exception if abnormal completion
                awtToolkit.getDefaultToolkit().beep()

                self.gui.startButton.enabled = True
                self.gui.cursor = None  #turn off the wait cursor
            except ExecutionException, e:
                awtToolkit.getDefaultToolkit().beep()

                self.gui.startButton.enabled = True
                self.gui.cursor = None  #turn off the wait cursor
                raise e.getCause()

###########################################################################
class Task2(SwingWorker):

      def __init__(self, gui):
            self.gui = gui
            SwingWorker.__init__(self)

      def doInBackground(self):


            def graficar(name,LabelA,A):
                  import org.jfree.data.xy.DefaultXYDataset as DefaultXYDataset
                  import org.jfree.chart.renderer.xy.XYSplineRenderer as
XYSplineRenderer
                  import org.jfree.chart.axis.NumberAxis as NumberAxis
                  import org.jfree.chart.plot.XYPlot as XYPlot
                  import org.jfree.chart.JFreeChart as JFreeChart
                  import org.jfree.chart.ChartFactory as ChartFactory
                  import org.jfree.chart.plot.PlotOrientation as PlotOrientation
                  import org.jfree.ui.RectangleInsets as RectangleInsets
                  import java.awt.Font as Font
                  from java.awt import geom,Color,BasicStroke
                  from org.jfree.chart.plot import CombinedDomainXYPlot
```

```
                from org.jfree.chart.axis import AxisLocation
                import org.jfree.chart.JFreeChart as JFreeChart
                import org.jfree.chart.renderer.xy.XYLineAndShapeRenderer as
XYLineAndShapeRenderer


                dataset = DefaultXYDataset()
                dataset.addSeries("Series1", A)
                #dataset1 = DefaultXYDataset()

                renderer = XYLineAndShapeRenderer()
                renderer.setSeriesStroke(0, BasicStroke(1))
                #xax = NumberAxis("x")
                #yax = NumberAxis("y")
                #plot = XYPlot(dataset,None,yax, spline)

                chart=ChartFactory.createXYLineChart(name,
                                           'Time [frame]',
                                           LabelA,
                                           dataset,
                                           PlotOrientation.VERTICAL,
                                           False,
                                           True,
                                           False)

                chart.getTitle().setFont( Font("Dialog", Font.BOLD, 30) )


                size =5.0
                delta = size / 2.0;
                shape1 = geom.Rectangle2D.Double(-delta, -delta, size, size)
                renderer.setSeriesShape(0, shape1)


                chart.setBackgroundPaint(Color.white)
                plot = chart.getPlot()
                plot.setBackgroundPaint(Color.white)
                plot.setRangeGridlinesVisible(True)
                plot.setAxisOffset(RectangleInsets.ZERO_INSETS)
                axis = plot.getRangeAxis()
                axis2 = plot.getDomainAxis()
                font = Font("Dialog", Font.BOLD, 20)
                axis2.setTickLabelFont(font)
                axis2.setLabelFont(font)
                axis.setLabelFont(font)
                axis.setTickLabelFont(font)
                axis.setLabelPaint(Color.blue)
                axis.setTickLabelPaint(Color.blue)
                axis2.setLabelPaint(Color.black)
                axis2.setTickLabelPaint(Color.black)
                  #plot.setRangeAxis(1, axis3)
                  #plot.setDataset(1,dataset1 )
                  #plot.mapDatasetToRangeAxis(1, 1)

                plot.setDomainGridlinesVisible(True)
```

```
            plot.setRangeGridlinesVisible(True)
            plot.setRangeGridlinePaint(Color.black)
            plot.setDomainGridlinePaint(Color.black)
          rangeAxis = plot.getRangeAxis()

rangeAxis.setStandardTickUnits(NumberAxis.createIntegerTickUnits())
          rangeAxis.setAutoRangeIncludesZero(False)
          #rangeAxis.setRange(0, maxY*1.10)
          plot.setRenderer(0,renderer)
          renderer.setSeriesPaint(0, Color.blue)
          bi = chart.createBufferedImage(1200, 800)
          imp =  ImagePlus("Results", bi)
          imp.show()




     ROI1=imp1.getRoi()
     print ROI1
     rect=ROI1.getBounds()
     print rect
     print rect.x
     print rect.y
     print rect.width
     print rect.height
     imp2.show()
     #imp2=WindowManager.getImage(WindowManager.getIDList()[0])
     xt= float(self.gui.xtrackField.text)
     yt= float(self.gui.ytrackField.text)
     #imp1 = WindowManager.getImage(WindowManager.getIDList()[1])
     cal=imp2.getCalibration()
     ip2=imp2.getProcessor()
     imgW=imp2.getWidth()
     imgH=imp2.getHeight()
     W=4
     H=4
     SL=imp2.getImageStackSize()
     A=ip2.getIntArray()
     xr=int(rect.x+rect.width/2.0)
     yr=int(rect.y+rect.height/2.0)

     def IMGmax(A,xr,yr):
          MAXV=[0]
          MAXloc=[]
          maximo=0
          for i in range(xr-5,xr+5):
               for j in range(yr-5,yr+5):
                    if A[i][j]>maximo:
                         maximo=A[i][j]
                         MAXloc.append([i,j])
                         #MAXV.append(max(A[i]))
                         #MAXloc.append([i,A[i].index(max(A[i]))])
          #print MAXV,MAXloc
          #print max(MAXV)
```

```
        #print MAXloc[MAXV.index(max(MAXV))]
        X=MAXloc[-1][0]
        Y=MAXloc[-1][1]
        return X,Y


    def Mean(A,x,y,W,H):
        mean=0
        for i in range(x,x+W):
            for j in range(y,y+H):
                mean=mean+A[i][j]
                #print i,j,A[i][j]
        return mean/(1.0*W*H)
    def optimize(A,X,Y,W,H):
        meansloc=[]
        means=[]
        for xi in range(W):
            for yi in range(H):
                #print X-xi,Y-yi
                means.append(Mean(A,X-xi,Y-yi,W,H))
                meansloc.append([X-xi,Y-yi])
        return meansloc[means.index(max(means))]
    IntDen=[]
    area=[]
    average=[]

    for i in range(1,SL+1,1):
        imp2.setSlice(i)
        ip2=imp2.getProcessor()
        A=ip2.getIntArray()
        X,Y=IMGmax(A,xr,yr)
        xr=X
        yr=Y
        ROI=ShapeRoi(Ellipse2D.Float(X-5/2.0,Y-5/2.0, 5, 5))

        #loc=optimize(A,X,Y,W,H)
        #ROI=Roi(loc[0],loc[1],W,H)
        print ROI
        imp2.setRoi(ROI)
        stats = imp2.getStatistics()
        IntDen.append(stats.mean*3.1415*5)
        area.append(stats.area)
        average.append(stats.mean)
    #print IntDen,area


    name=filename[:-4]+'_abl_region.txt'
    filepath1 = directory+name

    print filepath1,name
    o = open(filepath1,"wb") #open for write
    line='%header Ablation Tracker
\n%frame\tArea\tIntDen\tMeanGrayValue\n'
    o.write(line)
    XVA=range(1,len(area)+1)
```

```
            for i in range(len(area)):

      line=str(i+1)+'\t'+str(area[i])+'\t'+str(IntDen[i])+'\t'+str(average[i])+'
\n'
                  o.write(line)


            o.close()

            graficar('ablation ','Intensity[AU]',[XVA,IntDen])


            table1 = ResultsTable()
            roiM = RoiManager(True)
            pa = ParticleAnalyzer(ParticleAnalyzer.ADD_TO_MANAGER,
Measurements.AREA | Measurements.CENTROID| Measurements.ELLIPSE|
Measurements.CIRCULARITY| Measurements.SLICE| Measurements.RECT, table1, 8,
Double.POSITIVE_INFINITY, 0.0, 1.0)
            pa.setHideOutputImage(True)
            pa.setRoiManager(roiM)

            print 'flag1'
            for i in range(imp1.getStackSize()):
                  imp1.setSlice(i+1)
                  ip1=imp1.getProcessor()
                  ip1.setThreshold(200, 255, ImageProcessor.NO_LUT_UPDATE)
                  pa.analyze(imp1)
            a = table1.getColumn(0)
            frame = table1.getColumn(27)
            X = table1.getColumn(6)
            Y = table1.getColumn(7)
            Major = table1.getColumn(15)
            Minor = table1.getColumn(16)
            bx = table1.getColumn(11)
            by = table1.getColumn(12)
            angle = table1.getColumn(31)
            Circ = table1.getColumn(18)
            w= table1.getColumn(13)
            h = table1.getColumn(14)
            #print a,b
            cal=imp1.getCalibration()
            #xt=cal.getX(xt)
            #yt=cal.getY(yt)
            Tracklist=[]
            print 'flag2'

            '''
            for i in range(len(X)):

                  if xt+cal.getX(15)>X[i] and xt-cal.getX(15)<X[i] and yt-
cal.getY(15)<Y[i] and yt+cal.getY(15)>Y[i]:
                        Tracklist.append(i)
                        xt=X[i]
                        yt=Y[i]
            '''
```

```
            for i in range(len(X)):
                  if
ROI1.contains(int(cal.getRawX(X[i])),int(cal.getRawY(Y[i]))):
                        Tracklist.append(i)

            print Tracklist
            means = []
            print len(roiM.getRoisAsArray())
            for roi in roiM.getRoisAsArray():
                  imp2.setSlice(roi.getPosition())
                  imp2.setRoi(roi)
                  stats = imp2.getStatistics(Measurements.MEAN)
                  means.append(stats.mean)
            print len(means)

            name=filename[:-4]+'_nucleus_intensity.txt'
            filepath1 = directory+name
            print filepath1,name
            o = open(filepath1,"wb") #open for write
            line='%header Nucleus Tracker \n%i\tA\tRawIntDen\tMean\tX\tY\n'
            o.write(line)

            mframe=[]
            ma=[]
            mmeans=[]
            mX=[]
            mY=[]
            for i in Tracklist:
                  if frame.count(frame[i])>1:
                        if mframe[-1]<frame[i]:
                              mframe.append(frame[i])
                              ma.append(a[i])
                              mmeans.append(means[i])
                              mX.append(X[i])
                              mY.append(Y[i])
                        if mframe[-1]==frame[i]:
                              ma[-1]=ma[-1]+a[i]
                              mmeans[-1]=(mmeans[-1]+means[i])/2.0
                              mX[-1]=(mX[-1]+X[i])/2.0
                              mY[-1]=(mY[-1]+Y[i])/2.0
                  elif frame.count(frame[i])==1:
                        mframe.append(frame[i])
                        ma.append(a[i])
                        mmeans.append(means[i])
                        mX.append(X[i])
                        mY.append(Y[i])
            YVA=[]
            for i in range(len(mframe)):

      line=str(int(mframe[i]))+'\t'+str(round(ma[i],3))+'\t'+str(round(mmeans[i]
*ma[i]/(cal.getX(1)*cal.getY(1)),3))+'\t'+str(round(mmeans[i],3))+'\t'+str(round
(mX[i],3))+'\t'+str(round(mY[i],3))+'\n'
                  YVA.append(round(mmeans[i]*ma[i]/(cal.getX(1)*cal.getY(1)),3))
```

```
                o.write(line)


            o.close()
            print len(mframe),len(ma),len(mmeans),len(mX),len(mY)

            graficar('Nucleus','Intensity[AU]',[mframe,YVA])



      def done(self):
            try:
                self.get()  #raise exception if abnormal completion
                awtToolkit.getDefaultToolkit().beep()

                self.gui.startTrackButton.enabled = True
                self.gui.cursor = None  #turn off the wait cursor
            except ExecutionException, e:
                awtToolkit.getDefaultToolkit().beep()

                self.gui.startTrackButton.enabled = True
                self.gui.cursor = None  #turn off the wait cursor
                raise e.getCause()


###########################################################################


###########################################################################
class Task3(SwingWorker):

      def __init__(self, gui):
            self.gui = gui
            SwingWorker.__init__(self)

      def doInBackground(self):

            od = OpenDialog("Choose a file", None)
            global filename
            filename = od.getFileName()
            if filename is None:
                  print "User canceled the dialog!"
            else:
                  global directory
                  directory = od.getDirectory()
            filepath = directory + filename
            filepath2= directory + filename[:-4]+'Track'+filename[-4:]
            print "Selected file path:", filepath
            global imp1,imp2
            IJ.run("Bio-Formats Importer", "open=["+filepath+"] autoscale
color_mode=Colorized view=Hyperstack stack_order=XYCZT")
            imp2= WindowManager.getCurrentImage()
            IJ.run("Bio-Formats Importer", "open=["+filepath2+"] autoscale
color_mode=Colorized view=Hyperstack stack_order=XYCZT")
            imp1= WindowManager.getCurrentImage()
```

```
            cal=imp1.getCalibration()
            def doSomething(imp,loc):
                  """ A function to react to a mouse click on an image canvas.
"""
                  #print "clicked on: " ,imp
                  #print loc.getX(),loc.getY()
                  #print cal.getX(loc.getX()),cal.getX(loc.getY())
                  self.gui.xtrackField.text=str(cal.getX(loc.getX()))
                  self.gui.ytrackField.text=str(cal.getX(loc.getY()))
                  ip=imp.getProcessor()



                  if self.gui.checkbox.isSelected():
                        if ip.getPixel(int(loc.getX()),int(loc.getY()))>250:
                              ip.setColor(0)
                              ip.drawPixel(int(loc.getX()),int(loc.getY()))

      FileSaver(imp).saveAsTiffStack(directory+filename[:-4]+'Track'+filename[-
4:])
                        else:
                              ip.setColor(255)
                              ip.drawPixel(int(loc.getX()),int(loc.getY()))

      FileSaver(imp).saveAsTiffStack(directory+filename[:-4]+'Track'+filename[-
4:])
            class ML(MouseAdapter):
                  def mousePressed(self, event):
                        canvas = event.getSource()
                        imp = canvas.getImage()
                        loc= canvas.getCursorLoc()
                        doSomething(imp,loc)

            listener = ML()

            win = imp1.getWindow()
            win.getCanvas().addMouseListener(listener)

      def done(self):
            try:
                self.get()  #raise exception if abnormal completion
                awtToolkit.getDefaultToolkit().beep()

                self.gui.LoadTrackButton.enabled = True
                self.gui.cursor = None  #turn off the wait cursor
            except ExecutionException, e:
                awtToolkit.getDefaultToolkit().beep()

                self.gui.LoadTrackButton.enabled = True
                self.gui.cursor = None  #turn off the wait cursor
                raise e.getCause()


###########################################################################
```

```
###########################################################################
# Creating The GUI
###########################################################################
class KinetochoreTracker(JPanel, PropertyChangeListener):
    def __init__(self):
            JPanel.__init__(self, awtBorderLayout(),
                            border = BorderFactory.createEmptyBorder(20, 20, 20,
20)
                            )

            #Create the demo's UI.
            self.loadButton = JButton("Load", actionPerformed=self.loadPressed)
            self.startButton = JButton("Start",
actionPerformed=self.startPressed)
            self.ClearButton = JButton("Clear",
actionPerformed=self.clearPressed)
            self.startTrackButton = JButton("Start Track",
actionPerformed=self.startTrackPressed)
            self.LoadTrackButton = JButton("Load Track",
actionPerformed=self.LoadTrackPressed)
            self.progressBar = JProgressBar(0, 100, value=0, stringPainted=True)
            self.checkbox = JCheckBox( 'Editor Mode', selected=0,
itemStateChanged = self.cbTest );

            self.tresholdField = JTextField('100',15)
            self.HmField = JTextField('100',15)
            self.HrField = JTextField('1',15)
            self.tolField = JTextField('150',15)
            self.xtrackField = JTextField('0',15)
            self.ytrackField = JTextField('0',15)
            panel = JPanel(GridLayout(0,2))
            panel.add(self.loadButton)
            panel.add(JLabel("", SwingConstants.RIGHT))
            panel.add(self.startButton)
            panel.add(self.progressBar)
            panel.add(JLabel("Treshold:", SwingConstants.RIGHT))
            panel.add(self.tresholdField)
            panel.add(JLabel("HoodMean Treshold:", SwingConstants.RIGHT))
            panel.add(self.HmField)
            panel.add(JLabel("Hood radius:", SwingConstants.RIGHT))
            panel.add(self.HrField)
            panel.add(JLabel("Local Maxima Tolerance:", SwingConstants.RIGHT))
            panel.add(self.tolField)
            panel.add(self.startTrackButton)
            panel.add(self.LoadTrackButton)
            panel.add(JLabel("Aproximate X coordinate", SwingConstants.RIGHT))
            panel.add(self.xtrackField)
            panel.add(JLabel("Aproximate Y coordinate", SwingConstants.RIGHT))
            panel.add(self.ytrackField)
            panel.add(self.ClearButton)
            panel.add(JLabel("", SwingConstants.RIGHT))
            panel.add(self.checkbox)
            self.add(panel, awtBorderLayout.PAGE_START)
```

```
def cbTest( self, e ) :

        print self.checkbox.isSelected()

def startPressed(self, e):
        "Invoked when the user presses the start button"
        self.startButton.enabled = False
        self.cursor = awtCursor.getPredefinedCursor(awtCursor.WAIT_CURSOR)

        #Instances of javax.swing.SwingWorker are not reusuable, so
        #we create new instances as needed.
        task = Task(self)
        task.addPropertyChangeListener(self)
        task.execute()

def clearPressed(self, e):
        "Invoked when the user presses the start button"

        IJ.run("Close All")

        #Instances of javax.swing.SwingWorker are not reusuable, so
        #we create new instances as needed.

def loadPressed(self, e):
        self.loadButton.enabled = False
        self.cursor = awtCursor.getPredefinedCursor(awtCursor.WAIT_CURSOR)

        #Instances of javax.swing.SwingWorker are not reusuable, so
        #we create new instances as needed.
        load = Load(self)
        load.addPropertyChangeListener(self)
        load.execute()

def startTrackPressed(self, e):
        "Invoked when the user presses the start button"
        self.startTrackButton.enabled = False
        self.cursor = awtCursor.getPredefinedCursor(awtCursor.WAIT_CURSOR)

        #Instances of javax.swing.SwingWorker are not reusuable, so
        #we create new instances as needed.
        task2 = Task2(self)
        task2.addPropertyChangeListener(self)
        task2.execute()

def LoadTrackPressed(self, e):
        "Invoked when the user presses the start button"
        self.LoadTrackButton.enabled = False
        self.cursor = awtCursor.getPredefinedCursor(awtCursor.WAIT_CURSOR)

        #Instances of javax.swing.SwingWorker are not reusuable, so
        #we create new instances as needed.
        task3 = Task3(self)
        task3.addPropertyChangeListener(self)
        task3.execute()
```

```
    def propertyChange(self, e):
        # Invoked when task's progress property changes.
            if e.propertyName == "progress":
                  self.progressBar.value = e.newValue

def createAndShowGUI():
    # Create the GUI and show it. As with all GUI code, this must run
    # on the event-dispatching thread.
    frame = JFrame("Nucleus Tracker FinalRev",
                    defaultCloseOperation = JFrame.DISPOSE_ON_CLOSE,
                    )
    frame.setBackground(Color.GRAY)
    #Create and set up the content pane.
    newContentPane = KinetochoreTracker()
    newContentPane.setOpaque(True)      #content panes must be opaque
    frame.contentPane = newContentPane

    #Display the window.
    frame.pack()
    frame.visible = True

###########################################################################
# Running the plug-in
###########################################################################
class Runnable(Runnable):
    def __init__(self, runFunction):
        self._runFunction = runFunction


    def run(self):
        self._runFunction()

###########################################################################
if __name__ == '__main__':
    SwingUtilities.invokeLater(Runnable(createAndShowGUI))
```
